# Rapid Identification of Antifungal Compounds against *Exserohilum rostratum* Using High Throughput Drug Repurposing Screens

**DOI:** 10.1371/journal.pone.0070506

**Published:** 2013-08-21

**Authors:** Wei Sun, Yoon-Dong Park, Janyce A. Sugui, Annette Fothergill, Noel Southall, Paul Shinn, John C. McKew, Kyung J. Kwon-Chung, Wei Zheng, Peter R. Williamson

**Affiliations:** 1 National Center for Advancing Translational Sciences, National Institutes of Health, Bethesda, Maryland, United States of America; 2 Laboratory of Clinical Infectious Diseases, National Institute of Allergy and Infectious Diseases, National Institutes of Health, Bethesda, Maryland, United States of America; 3 University of Texas Health Science Center, San Antonio, Texas, United States of America; 4 Section of Infectious Diseases, Immunology and International Medicine, University of Illinois College of Medicine, Chicago, Illinois, United States of America; Research Institute for Children and the Louisiana State University Health Sciences Center, United States of America

## Abstract

A recent large outbreak of fungal infections by *Exserohilum rostratum* from contaminated compounding solutions has highlighted the need to rapidly screen available pharmaceuticals that could be useful in therapy. The present study utilized two newly-developed high throughput assays to screen approved drugs and pharmaceutically active compounds for identification of potential antifungal agents. Several known drugs were found that have potent effects against *E. rostratum* including the triazole antifungal posaconazole. Posaconazole is likely to be effective against infections involving septic joints and may provide an alternative for refractory central nervous system infections. The anti-*E. rostratum* activities of several other drugs including bithionol (an anti-parasitic drug), tacrolimus (an immunosuppressive agent) and floxuridine (an antimetabolite) were also identified from the drug repurposing screens. In addition, activities of other potential antifungal agents against *E. rostratum* were excluded, which may avoid unnecessary therapeutic trials and reveals the limited therapeutic alternatives for this outbreak. In summary, this study has demonstrated that drug repurposing screens can be quickly conducted within a useful time-frame. This would allow clinical implementation of identified alternative therapeutics and should be considered as part of the initial public health response to new outbreaks or rapidly-emerging microbial pathogens.

## Introduction

Unusual or highly antibiotic resistant organisms may subject large numbers of individuals to unexpected infectious diseases due to greater globalization that brings more widespread distribution networks and potential threats such as bioterrorism. Limited therapeutic options or failures in conventional therapy during these outbreaks can be encountered because of either intolerable drug toxicities or lack of efficacious drugs. Recently, a large outbreak of fungal infections has been caused by the widespread distribution of contaminated preservative-free methylprednisolone acetate prepared by a single compounding pharmacy [1], [2], [3], [4]. It has currently resulted in 741 infections with 55 deaths [5]. *Exserohilum rostratum*, a dermatiaceous fungus commonly found on plants, in soil and in households, has been identified as one of the predominant pathogens in the current multistate outbreak of fungal meningitis and other infections associated with contaminated steroid injections. Although it rarely causes infections in healthy people, infections of skin and corneal tissues as well as more disseminated infections in immunocompromised populations have been reported [6], [7], [8].

*E. rostratum* is sensitive to amphotericin B, a commonly used antifungal agent, but the severe and potentially lethal side-effects of this drug have limited its use in certain patients. While traditional antibiotic susceptibility testing has provided initial recommendations of using amphotericin B for treatment, the advanced age (median 69) of the patient population in this outbreak has limited the therapeutic efficacy in many patients, mainly due to drug toxicity. There are few alternative drugs that are known for the treatment of infections caused by *E. rostratum*. The conventional process for drug discovery and development cannot accommodate such a sudden outbreak as it requires 10–12 years in average to develop a drug. Recently, a drug repurposing screen with approved drugs and pharmacologically active agents [9] has emerged as an alternative approach to rapidly identify new therapeutic indications that have been successfully applied to several diseases [10], [11], [12], [13], [14], [15]. This unique approach may also help to quickly identify alternative therapeutics for the treatment of infections caused by outbreaks such as that by *E. rostratum*.

Here we report the development and optimization of two assays using *E. rostratum* hyphae and conidia in an ATP content assay format for high throughput screening. Both assays were screened in parallel against two known compound libraries including 4096 approved drugs and 1280 compounds with pharmacologically known activities. Within seven weeks, the activities of 20 known antifungals, 8 other anti-infectious agents and 10 other drugs against *E. rostratum* were identified from the screens. While some of these drugs may be considered as alternative therapeutics to treat *E. rostratum* infections, others could serve as tools for identification of new molecular targets for future drug development.

## Materials and Methods

### Materials

Amphotericin B (catalog # A9528) was purchased from Sigma-Aldrich (St. Louis, MO). The ATP content kit (ATPlite, catalog No. 6016941) was purchased from PerkinElmer (Waltham, MA). PBS (Catalog No. 10010049) was purchased from Life technologies. The 1536-well white sterile tissue culture treated polystyrene plates (Catalog No. 789092-F) were purchased from Greiner Bio-One (Monroe, NC).

### Preparation of *Exserohilum rostratum* conidia and hyphal fragments

Conidia and hyphae of *E. rostratum* were obtained as described by Richard et al. [16], with the following modifications. Briefly, conidia were harvested from Potato Dextrose Agar (PDA) cultured media with 0.05% Tween 80, and the conidial suspension was filtered using a Cell Strainer (100 µm, BD Falcon REF 352340). After centrifugation at 700*g* for 10 min, the suspension was decanted and conidia were resuspended at 1×10^5^ per ml in RPMI and counted in a hemocytometer. Hyphae were harvested from yeast extract peptone dextrose (YPD) culture media with 0.05% Tween 80. Hyphal fragments were sized by vortexing 15 sec twice with 0.4 mm glassbeads, and the hyphae suspension was filtered by cheese cloth twice. Microscopy was used to determine the size of hyphal fragments, which ranged between 10–50 µm. To normalize concentrations of hyphal fragments for batch to batch consistency, carbohydrate analysis was performed by a phenol-sulfuric acid method as previously described [17]. The final stock concentration of hyphae was adjusted to 1.0 (OD_490_) per 100 µl.

### Mammalian cell culture

Human neuroblastoma SH-SY5Y cell line (Catalog No. CRL-2266) was purchased from ATCC (Manassas, VA). SH-SY5Y cell line was cultured in 175-cm^2^ tissue culture flasks (Costar, Cambridge, MA) with 30 ml of growth medium at 37°C in a 5% CO_2_ humidified atmosphere. Growth medium was made with Dulbecco's Modified Eagle Medium: Nutrient Mixture F-12 with 10% fetal bovine serum (FBS). Growth medium was replaced every other day and cells were passed at 75% confluence.

### ATP content assay

The ATPLite assay kit, consisting of luciferase/luciferin and detergent, was used to quantitate cellular ATP levels as a marker for cell viability (Table 1). For assay development, hyphal fragments or conidia in PBS were plated in a seeding density of 3,750, 7,500 and 15,000 cellular particles/well with a final volume of 5 µl/well using the Multidrop-Combi dispenser in white 1536-well plates. Conidia or hyphae fragments were incubated for 24 h at 37°C in a 5% CO_2_ humidified atmosphere. ATPLite detection reagents were added at 4 µl/well. The assay plates were incubated at room temperature for 15 minutes before reading luminescence intensity on a ViewLux plate reader (Perkin Elmer, Waltham, MA). Conidia suspended in RPMI medium were inoculated at concentrations of 62.5, 125 and 250 conidia/well with a final volume of 5 µl/well in white 1536-well plates. The conidia assay was developed and optimized in the same way as the hyphal assay.

**Table 1:** Assay protocol for the measurements of fungicidal compounds.

Step	Parameter	Value	Description
1	PBS/Medium	2.5 µl/well	PBS or RPMI Medium
2	Compound	0.023 µl/well	Compound in DMSO solution
3	Hyphae/Conidia	2.5 µl/well	Hyphae in PBS or Conidia in RPMI Medium
4	Incubation	24 hr	37°C, 5% CO_2_
5	Detection reagent	4 µl/well	ATP content assay reagent
6	Incubation	15 min	Room temperature
7	Plate reading	Luminescence mode	ViewLux plate reader

Note: PBS was dispensed in the hyphae assay following by transferring of compound and addition of hyphae cell suspension. RPMI Medium, compound and conidia cells in RPMI Medium were sequentially added in the conidia assays.

### Compound library and liquid handling instrument

The library of pharmacologically active compounds (LOPAC) consists of 1280 small molecules with characterized biological activities. The LOPAC library (Sigma-Aldrich) has been extensively used for HTS assay validations [10], [18]. The NIH Chemical Genomics Center Pharmaceutical (NPC) collection was constructed in-house through a combined effort of compound purchasing and custom synthesis [9] and recently expanded to 4096 compounds. In this drug libarary, 52% of which are drugs approved for human or animal use by the United States Food and Drug Administration (FDA), 22% are drugs approved in Europe, Canada, or Japan, and the remaining 25% are compounds that have entered clinical trials or are research compounds commonly used in biomedical research. Compounds from both libraries were obtained as powder samples and dissolved in DMSO as 10 mM stock solutions, except several hundred from the NPC library that were prepared as 4.47 mM stock solutions due to solubility limitations. 2.5 µl/well PBS was dispensed into 1536-well plates using a Multidrop-Combi dispenser. Compound in DMSO solution was transferred in a volume of 23 nl/well using a NX-TR pintool station (WAKO Scientific Solutions, San Diego, CA). This additional PBS step for compound addition was designed to prevent the potential fungal contamination of compound libraries. The hyphal fragments in PBS were then added at 2.5 µl/well for a final seeding density of 15,000 hyphal fragments/well using the Multidrop-Combi dispenser. The final compound concentration was 46 µM in the primary screen. The assay plates were incubated for 24 h at 30°C before the detection of cellular viability with the ATP content assay. Screen of conidia was performed in a similar way except that PBS was replaced with RPMI medium and conidia were plated for a final seeding density of 250 conidia/well.

### Colony formation

10 µl of hyphal suspension (processed as described above) was transferred to 1.7 mL microfuge tubes, each containing 28 nM amphotericin B, 242 nM posaconazole, or 10% DMSO in 1 ml PBS. Tubes containing hyphae and drug were incubated at 37°C in a shaking incubator for 24 h, then 200 µl of the hyphae drug suspension was spread on Yeast Extract Peptone Dextrose (YPD) plates for 24 h at 30°C and the number of colony forming units were scored. The experiments were performed in triplicate.

### Data analysis

The primary screen data and curve fitting were analyzed using software developed internally at the NIH Chemical Genomics Center (NCGC) [19]. Total signals for cellular viability (100% cellular viability) were calculated from the wells with DMSO solvent and the complete cell killing (0% cellular viability) was calculated from 46 µM amphotericin B treated wells. IC_50_ values of compound confirmation data were calculated using the Prism software (Graphpad Software, Inc. San Diego, CA). All values were expressed as the mean +/− SD (n≥3).

## Results

### Development of fungicidal assays for hyphal fragments and conidia

**Figure 1:**
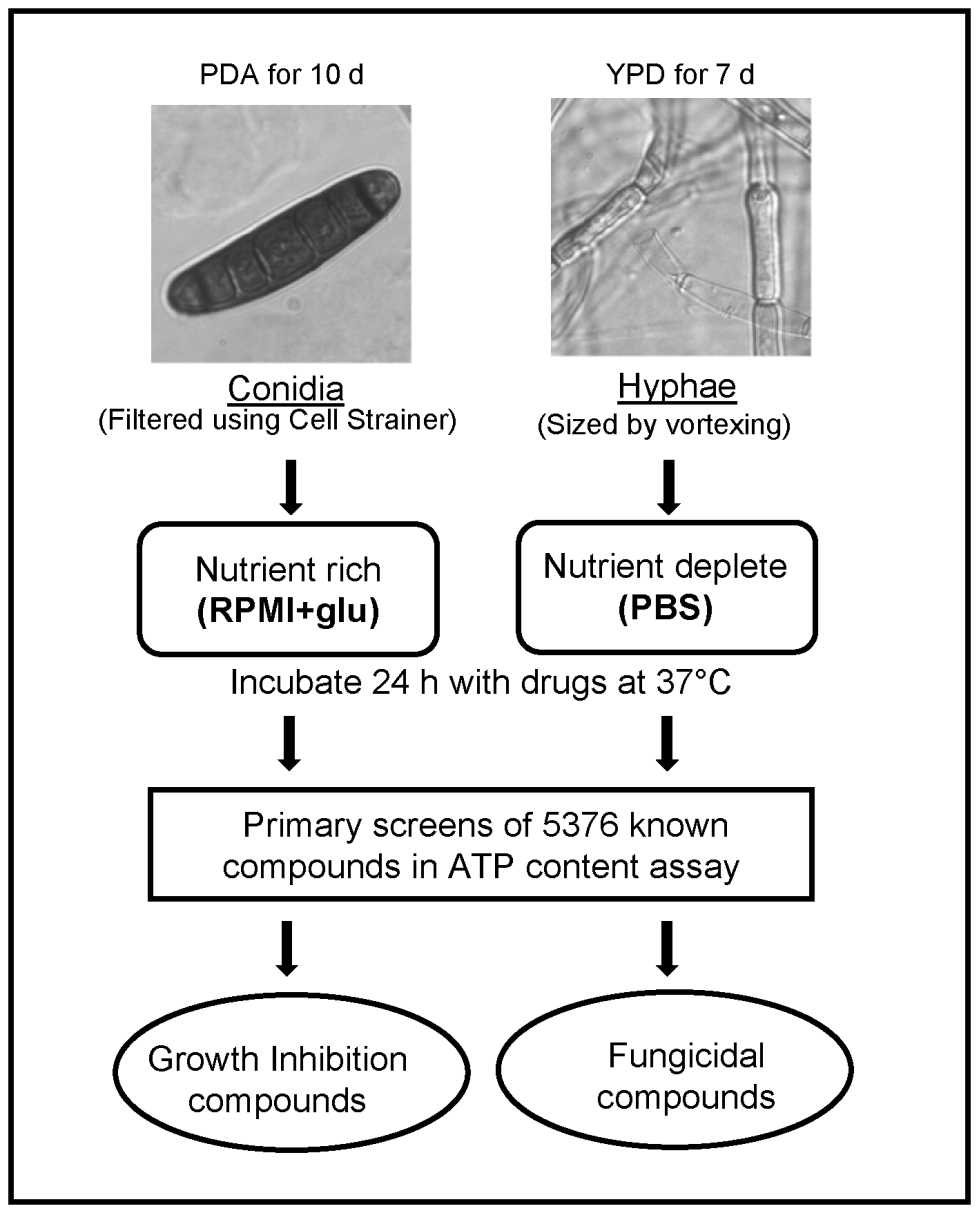
Scheme of hyphae and conidia assay methodology. Conidia harvested from colonies grown on potato dextrose agar (PDA) were incubated in nutrient-rich RPMI media with compounds to identify inhibitors of *E. rostratum* growth. Alternatively, hyphal fragments, obtained by growth on Yeast Extract Peptone Dextrose agar (YPD) and size-reduced by sonication, were incubated with compounds under nutrient-deprived conditions to identify fungicidal compounds.

In standard fungal growth inhibition assays, conidial suspensions are typically used to measure compound activities on the inhibition of fungal growth in a nutrient-rich RPMI medium [20]. Thus, the growth inhibition assay in RPMI medium was adapted for high-throughput screening to identify antifungal compounds (Fig. 1). However, fungal infections may also occur within nutrient-deprived environments such as in the central nervous system or abscess cavities [21]. In addition, many clinical data have indicated that the fungicidal activity of antifungal drugs, exemplified by the polyene amphotericin B, may be more important to clinical success for the treatments of fungal infections [22]. Thus, in addition to the standard growth inhibition assay using conidia, we also developed a hyphal fungicidal assay using nutrient-depleted medium for this drug repurposing screen (Fig. 1). In this study, both hyphae and conidia assays were used for a comprehensive evaluation of therapeutic potentials of antifungal drugs since the current outbreak of infections has been reported in both the CNS and within other closed spaces such as peripheral joints [3].

**Figure 2:**
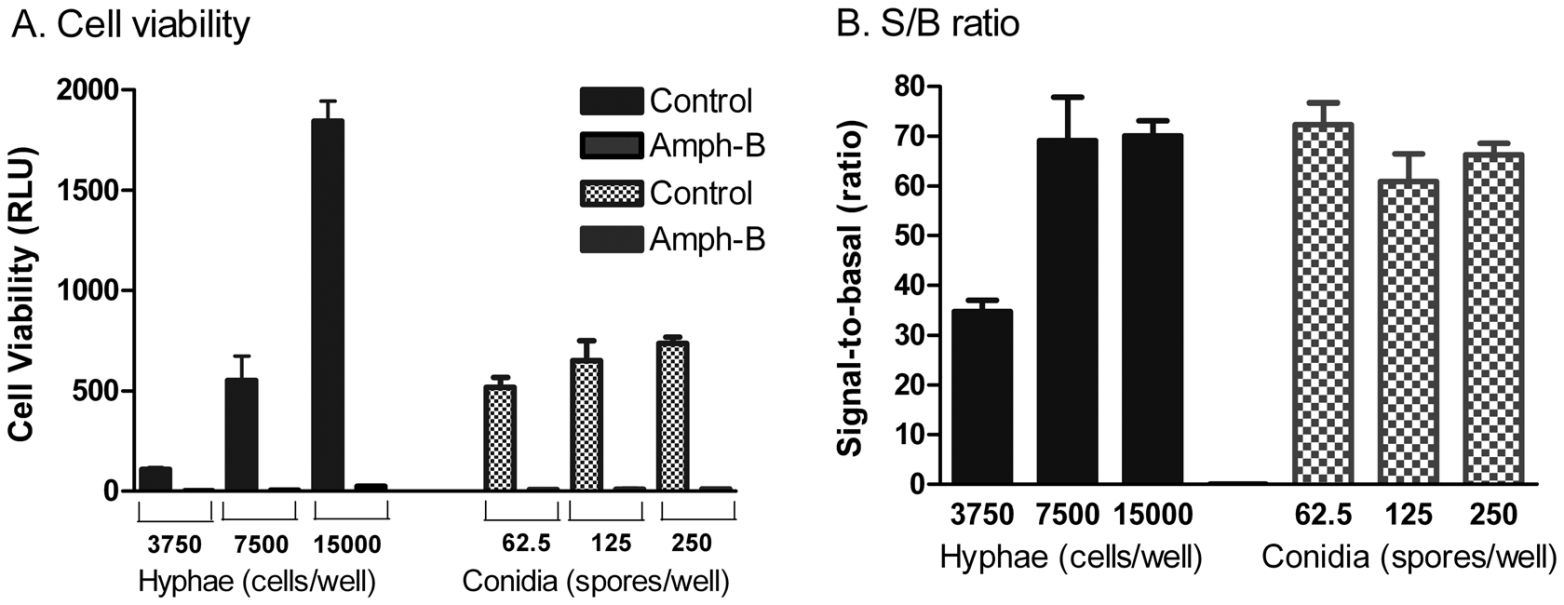
*E. rostratum* cell density optimization in a 1536-well plate assay format. (**A**) The viability of hyphal fragments was assayed at 3750, 7500 and 15000 fragments/well suspended in PBS after incubation with 46 µM amphotericin B (Amph-B) or DMSO (solvent control) for 24 h at 37°C using the ATP content assay. Similarly, the viability of conidia (spores) was assayed at 62.5, 125 and 250 cells/well in nutrition rich RPMI medium. (**B**) Signal-to-basal ratios of the hyphal fragment and conidia assays in different cell densities. The total signal and basal signal were defined by the cells treated with DMSO and 46 µM Amph-B, respectively. (RLU: relative luminescence units).

**Figure 3:**
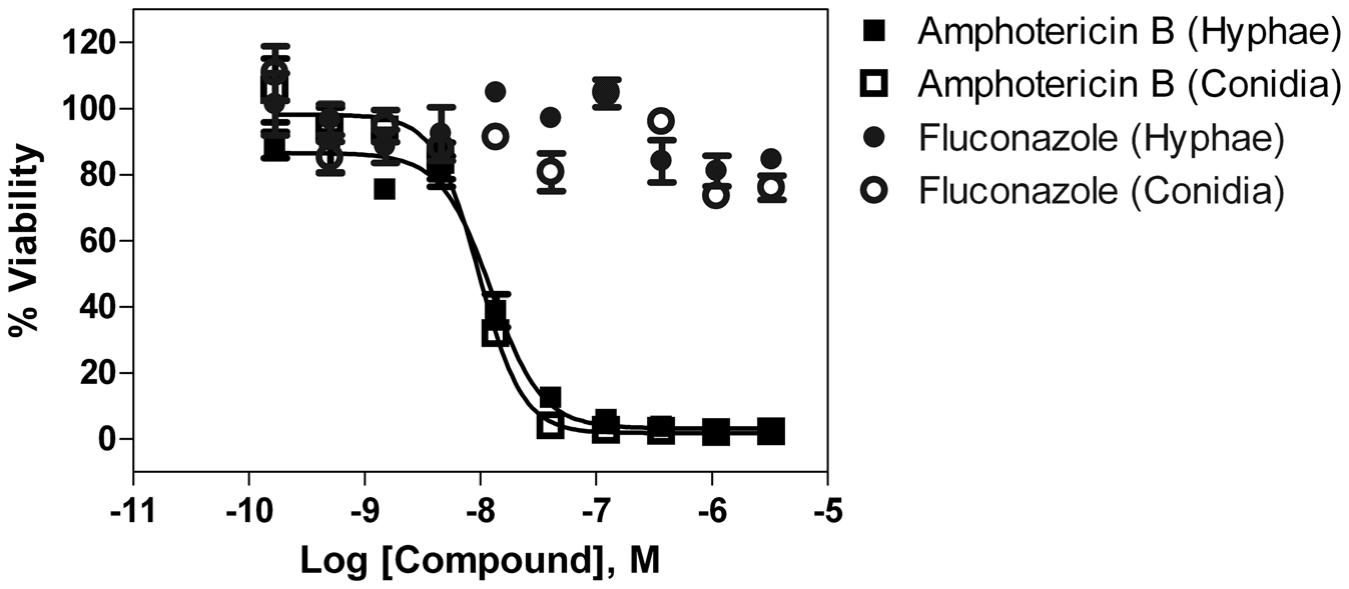
Concentration-response curves of Amphotericin B and fluconazole against *E. rostratum* in hyphal fragment and conidia assays. The IC_50_ values of amphotericin B in the hyphae assay with 15000 fragments/well and conidia assay with 250 cells/well were 12.4 nM (9.93 to 15.6 nM, 95% confidence intervals) and 9.71 nM (8.24 to 11.4 nM), respectively. Fluconazole showed no activity in both assays.

We used a commercially available ATP content assay kit to measure the viability of hyphal fragments or conidia after treatment with compounds. The bioluminescence signals are generated by the interaction of ATP in lysates of live cellular elements with luciferase and its substrate luciferin added from the assay kit. This assay was suitable for use in 1536-well plates because of its high sensitivity and simple assay procedure. The hyphal fragment density was first optimized using 3750, 7500 and 15000 fragments/well in 1536-well plates with amphotericin B as a positive control. The total ATP content signals in control group (without compound treatment) increased with the increase in hyphal densities while the signals in the group treated with amphotericin B for 24 hours reduced dramatically, indicating almost complete killing of hyphae (Fig. 2a). The signal-to-basal (S/B) ratio increased from 35 fold to 70 fold when the cellular densities rose from 3500 to 7500–15000 cellular equivalents/well (Fig. 2b). The hyphal fragment cellular density of 15000/well was then selected for further experiments. The conidia density was also optimized in a similar manner. The signals of viable conidia increased with increased cellular densities. We selected 250 conidia/well for subsequent experiments. The IC_50_ values of amphotericin B were similar in both our 1536-well plate assays (Fig. 3); 12.4 nM in the hyphal assay and 9.41 nM in the conidia assay that were 5 to 6 fold more potent than 60 nM determined from the microdilution method for filamentous fungi [23]. This discrepancy might be due to differences in assay formats as one measures the ATP contents in viable cellular elements and the microdilution method counts viable cell density directly. Fluconazole, another antifungal drug without activity against molds such as *E. rostratum*, showed no activity at concentrations up to 46 µM in either the hyphae or conidial assay format (Fig. 3). The results indicate that both hyphae and conidia assays in the ATP content assay format are suitable for compound screening to identify anti-*E. rostratum* agents.

### Compound library screening and hit confirmation

**Figure 4:**
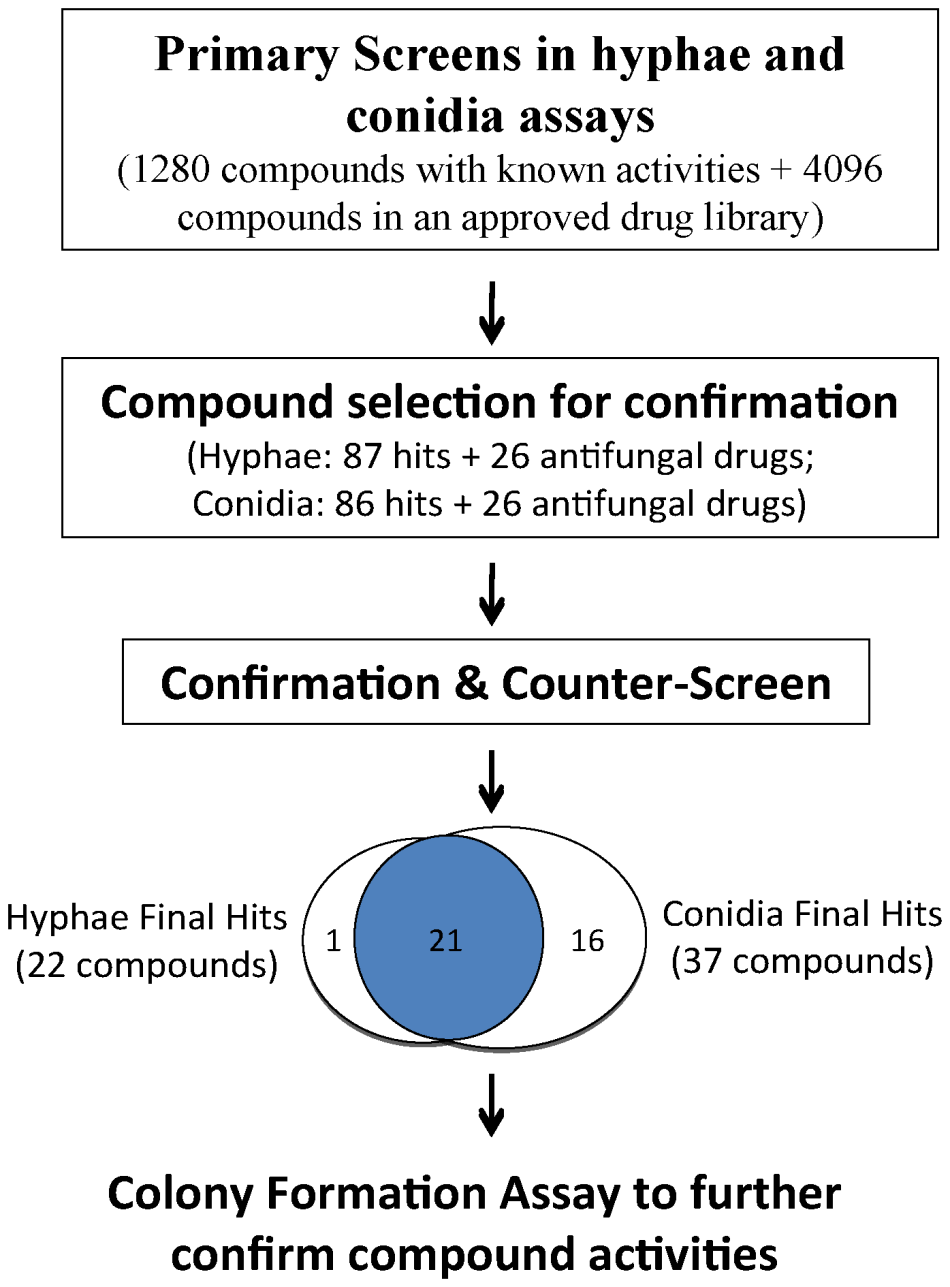
Flowchart of *E. rostratum* viability screens and compound confirmation in hyphal fragment (left) and conidia (right) assays. The primary screens of the LOPAC and approved drug libraries were carried out in the hyphal fragment and conidia assays. A group of 87 hits from the hyphae screen and a group of 86 hits from the conidia screen were selected for confirmation in the same assays along with an additional 26 antifungal drugs. A counter-screen using a mammalian SH-SY5Y cell line as a general cytotoxicity assay was also performed in parallel with the confirmation assays to determine the selectivity of these compounds against *E. rostratum*. The anti-*E. rostratum* activities of 22 compounds were confirmed in the hyphae assay and 37 compounds were confirmed in the conidia assay, with 22 of them being active in both assays. Eight selected compounds from the confirmed compounds were further tested in the traditional colony formation experiment to confirm their anti-*E. rostratum* activities.

Two small known compound libraries including the LOPAC (1280 compounds) and approved drugs (4096 compounds) were screened in parallel using the above optimized assays for hyphae and conidia in 1536-well plates (Fig. 4). A total of 87 primary hits were identified from the hyphal assay and 86 compounds were found from the conidia screen. These hits were retested in the same assays for their concentration response curves along with 26 additional known antifungal drugs (Table S1). A mammalian SH-SY5Y cell line was used in parallel as a counter-screen with the same ATP content assay kit to assess the specificity of these compounds for *E. rostratum*. As a result, 22 compounds in the hyphal assay and 37 compounds in the conidial assay showed activity against *E. rostratum* with IC_50_ values under 10 µM and with greater than 10-fold selectivity over the SH-SY5Y cells (Table 2). Among these confirmed compounds, 21 of them were active in both hyphal and conidia assays along with one compound active in the hyphal assay only and 16 compounds selective to the conidia assay.

**Table 2:** Confirmed active compounds against *E. rostratum*.

Compound Name	Hyphae		Conidia		SH-SY5Y		Reported drug activity	Reported mechanism of action
	IC_50_ (µM)	% Max. Resp.	IC_50_ (µM)	% Max. Resp.	% Max. Resp.	% Max. Resp.		
Known antifungals:								
Amphotericin B	0.014	84	0.012	104	inact.	inact.	Antifungal	Ergosterol synthesis
Posaconazole	0.121	78	0.097	106	inact.	inact.	Triazole antifungal	Ergosterol synthesis
Lanoconazole	0.203	65	0.38	103	inact.	inact.	Topical antifungal & antiparasite	Ergosterol synthesis
Azoxystrobin	0.198	81	0.304	83	inact.	inact.	Fungicide in agriculture	Respiration
Enilconazole	0.312	73	1.72	102	inact.	inact.	Fungicide in agriculture	Ergosterol synthesis
Captan	1.17	82	inact.	inact.	18.6	100	Phthalimide fungicide	Respiration
Itraconazole	2.19	80	1.24	100	inact.	inact.	Triazole antifungal	Ergosterol synthesis
Voriconazole	2.6	94	2.9	101	inact.	inact.	Triazole antifungal	Ergosterol synthesis
Ketoconazole	2.38	74	3.47	101	inact.	inact.	Broad spectrum antifungal	Ergosterol synthesis
Myriocin	2.75	81	13.6	92	inact.	inact.	Antifungal and immunosuppressive	Serine palmitoyltransferase
Amorolfine	4.51	81	7.86	102	inact.	inact.	Morpholine antifungal	Ergosterol synthesis
Pyrrolnitrin	5.59	80	0.409	101	inact.	inact.	Antifungal antibiotic	Respiration
Triticonazole	5.82	74	6.95	100	inact.	inact.	Fungicide	Ergosterol synthesis
Tioconazole	5.97	100	8.55	101	inact.	inact.	Antifungal medication	Ergosterol synthesis
Terconazole	6.12	100	9.06	101	inact.	inact.	Antifungal medication	Ergosterol synthesis
Luliconazole	6.4	82	4.39	100	inact.	inact.	Topical antifungal	Ergosterol synthesis
Thimerosal	inact.	inact.	0.139	102	2.63	96	Topic antiseptic and antifungal	Organomercury
Phenylmercuric acetate	inact.	inact.	0.146	99	0.417	96	Fungicide	Organomercury
Clioquinol	inact.	inact.	0.975	103	37.2	60	Antifungal and antiprotozoal	Not clear
Hexachlorophene	inact.	inact.	3.97	109	inact.	inact.	Disinfectant and fungicide	Respiration
Fenticlor	inact.	inact.	5.72	107	inact.	inact.	Topical antibacterial and antifungal	Not clear
Anti-infectious agents:								
Pentamidine isethionate	0.468	82	1.11	108	inact.	inact.	Antiprotozoal	No clear
Dequalinium dichloride	0.673	93	2.54	109	inact.	inact.	Topical bacteriostat	Not clear
Broxyquinoline	4.51	96	1.6	98	58.9	100	Anti-infective	Not clear
Calcimycin	inact.	inact.	0.735	51	3.95	94	Ionophorous, polyether antibiotic	Ionophore
Phenylmercuric borate	inact.	inact.	0.149	103	0.417	99	Topical antiseptic and disinfectant	Organomercury
Bithionol	inact.	inact.	1.87	100	inact.	inact.	Halogenated anti-infective	NF-κB inhibitor
Bleomycin sulfate	inact.	inact.	1.24	98	inact.	inact.	Glycopeptide antibiotic	DNA synthesis
Broquinaldol	inact.	inact.	7.75	101	inact.	inact.	Antimicrobial	Not clear
Other drugs:								
Tacrolimus	0.032	83	0.024	101	inact.	inact.	Immunosuppressive	Calcineurin phosphatase
Floxuridine	0.846	77	0.288	29	inact.	inact.	Antineoplastic antimetabolite	DNA synthesis
Oxaliplatin	0.935	76	4.95	95	inact.	inact.	Antineoplastic	DNA synthesis
Cerivastatin sodium	1.61	79	0.472	101	inact.	inact.	Hydroxymethylglutaryl-CoA Reductase Inhibitor	HMG-CoA reductase
Tetradonium bromide	inact.	inact.	2.44	103	inact.	inact.	Detergents and surface-active	Not clear
Sulfadiazine silver	inact.	inact.	1.3	98	26.3	87	Topical antibacterial	Not clear
Tribromosalan	inact.	inact.	1.93	99	inact.	inact.	N/A	NF-κB inhibitor
Iodoquinol	inact.	inact.	2.75	99	26.3	58	Intestinal antiseptic	Not clear
Tyrphostin AG 879	inact.	inact.	6.16	92	inact.	inact.	Inhibitors of nerve growth factor (NGF) TrkA	TRK receptor
Tyrphostin A9	inact.	inact.	1.98	100	18.6	61	Receptor tyrosine kinase inhibitor	CRAC channels

Note: % Max. Resp.: % maximal response (46 µM Amphotericin B is considered as 100%); Inact. in the conidia assay or SH-SY5Y cytotoxicity assay: no significant activity at the highest compound concentration (46 µM); Inact. in the hyphae assay - no significant activity in the primary screen.

**Figure 5:**
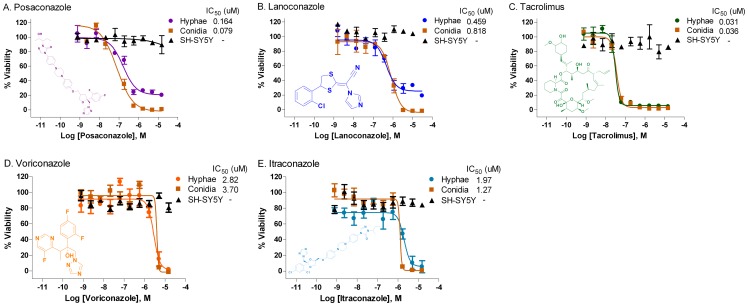
Representative confirmed compounds against *E. rostratum*. The antifungal activities of five compounds including (A) posaconazole, (B) lanoconazole, (C) tacrolimus, (D) voriconazole, and (E) itraconazole were compared in the hyphae and conidia assays with their cytotoxicities in SH-SY5Y mammalian cells. All of these five compounds exhibited similar antifungal activities in both hyphae and conidia assays with no significant cytotoxicity in the mammalian cells at the concentration up to 46 µM.

Several triazole antifungal drugs were among the confirmed compounds, including the most potent one – posaconazole (hyphae IC_50_ = 121 nM, conidia IC_50_ = 97 nM), as well as lanoconazole (hyphae IC_50_ = 203 nM, conidia IC_50_ = 380 nM), voriconazole (hyphae IC_50_ = 2.64 µM, conidia IC_50_ = 2.91 µM), itraconazole (hyphae IC_50_ = 2.19 µM, conidia IC_50_ = 1.24 µM), and luliconazole (hyphae IC_50_ = 6.40 µM, conidia IC_50_ = 4.39 µM) (Fig. 5, Table 2). The IC_50_ values were similar to these (posaconazole: 0.25 µg/ml or 360 nM; voriconazole: 2 µg/ml or 5.7 µM) determined using the microdilution method (Clinical and Laboratory Standards Institute) for filamentous fungi M38-A2 for this isolate [23].

**Figure 6:**
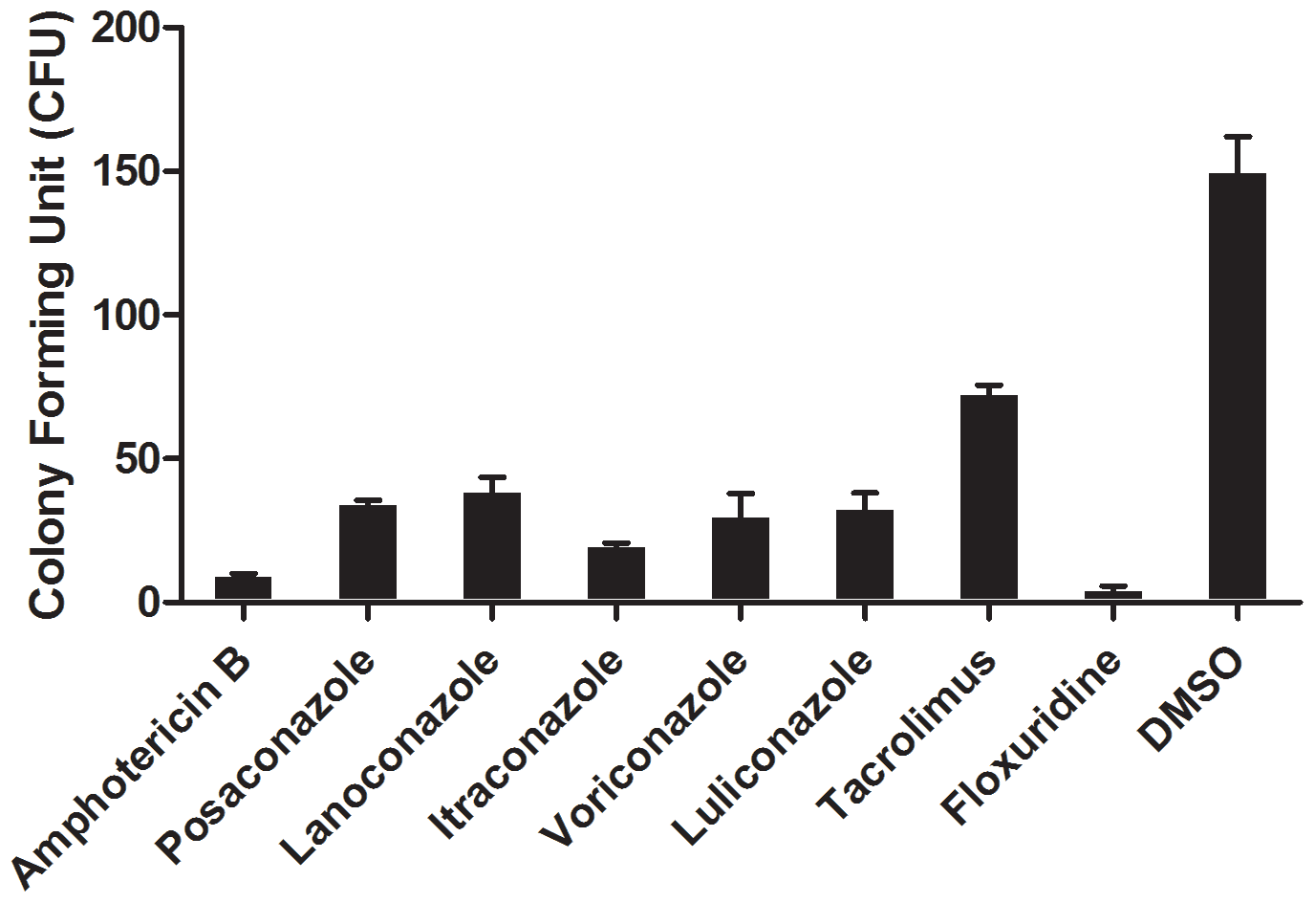
Distribution of molecular targets of 18 confirmed compoundss. The antifungal activities against *E. rostratum* were identified for 8 other anti-infectious agents and 10 other drugs (for details, see Table 2). Their molecular targets and mechanism of action are known although their antifungal activities were not previously reported. These reported molecular targets may implicate potential new directions for anti-*E. rostratum* drug development.

In addition to known antifungals, several other known drugs were identified as potential anti-*E. rostratum* agents. Bithionol, an old anti-parasitic drug with central nervous system penetration, was active with an IC_50_ of 1.87 µM in the conidia assay (Table 2). In addition, a number of known drugs with immunosuppressive or anti-cancer properties were also found having anti-*E. rostratum* acitivies, though they may not be suitable for primary therapy as they could suppress the immune response of patients. However, these drugs may provide prophylactic activity against *E. rostratum* infection if the patient was on these therapies for other reasons. For example, tacrolimus (Fig. 5D), an immunosuppressive agent used after organ transplantation, exhibited potent activity in both hyphae (IC_50_ = 32 nM) and conidia (IC_50_ = 24 nM) assays. Another known drug with anti- *E. rostratum* activity was floxuridine (Table 2), an antimetabolite used to treat a variety of tumors including brain tumors [24]. Its IC_50_ value in the hyphal assay was 846 nM. Floxuridine crosses the blood brain barrier with brain concentrations above 600 nM 8 hours after an intravenous (iv) administration of 20 mg/kg of the drug [25]. Among the 38 confirmed hits (Table 2), many may not be suitable for direct use to treat infections with *E. rostratum*. Diverse molecular targets and mechanism of action (Fig. 6) have been reported for the 8 ‘anti-infective agents’ and 10 ‘other agents’ listed in Table 2. Taken together, we have identified several non-antifungal drugs that exhibited fungicidal activity against *E. rostratum*. Since most are approved drugs, they may be used in either single use or in a combination with amphotericin B for the treatment or concomitant prophylaxis of infections caused by *E. rostratum*.

### Confirmation of fungicidal activity in colony formation assays

**Figure 7:**
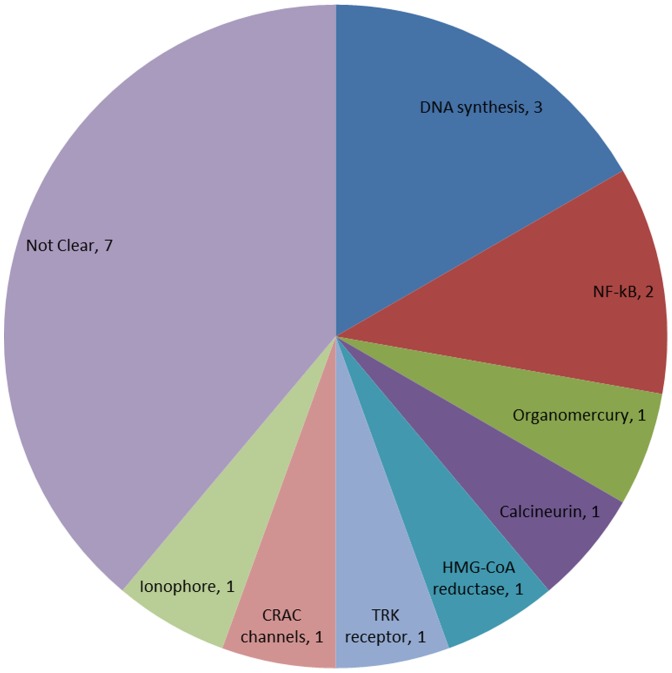
Validation of anti-*E. rostratum* activities of 8 confirmed compounds in colony forming assay. Hyphal fragment suspensions were treated with either 28 nM amphotericin B, 242 nM posaconazole, 406 nM lanoconazole, 4.38 µM itraconazole, 5.2 µM voriconazole, 12.8 µM luliconazole, 64 nM tacrolimus, 1.692 µM floxuridine or 1% DMSO (a solvent control) in PBS. Mixtures were cultured at 37°C in a shaking incubator for 24 h, then the hyphal suspensions were inoculated on Yeast Extract Peptone Dextrose (YPD) plates that were incubated for 1 day. The number of colonies on each YPD plate was counted. Results from three independent experiments were averaged; error bars show standard deviation.

To further validate the fungicidal activities of compounds identified from our new assays, we measured viability by colony forming units of *E. rostratum* hyphal fragments in the presence of amphotericin B, posaconazole, lanoconazole, voriconazole, itraconazole, luliconazole, tacrolimus and floxuridine (Fig. 7). After treatment with these compounds at 37°C for 24 h, all the compounds at concentrations of 2 times their respective IC_50_ values significantly reduced hyphae-derived colony forming units (CFU) compared to that in the DMSO control (p<0.01). Together, the results further demonstrate the activities of these compounds against *E. rostratum*.

## Discussion

The *E. rostratum* infection outbreak thus far has resulted in approximately 741 cases with a large number of CNS infections and 55 deaths. Advanced age of the patient population has limited therapeutic applications in some patients and many have responded suboptimally, requiring changes in therapy. The present pilot screen successfully identified several known drugs as alternative therapeutic choices from a collection of known drugs and pharmaceutically active compounds within a seven-week period. The original goal of such a screen was to identify a spectrum of antifungal agents such as posaconazole that could be used immediately to treat patients with *E. rostratum* infection. We also found a group of novel agents such as bithionol that could be developed further in animal models, preclinical and clinical studies in the event the outbreak continued or drug resistance was encountered. The results from this drug repurposing screen were also helpful in excluding agents that are unlikely to be useful in clinical therapy. For example, albendazole has shown activity against other molds such as *Aspergillus* [26] but is not active against *E. rostratum* in our experiments. Therefore, this study demonstrates that such a drug repurposing screen can be conducted within a useful time-frame and should be considered as part of the initial public health response to new outbreaks or emerging microbial pathogens.

To improve assessment of the *in vitro* activity of antifungal agents against hyphae, quantitative colorimetric assays using 2,3-bis(2-methoxy-4-nitro-5-[(sulphenylamino)carbonyl]-2H-tetrazoliumhydroxide (XTT) and a fluorometric assay using AlarmaBlue have been developed [27]. However, the lower signal-to-basal ratio in either assay limits their applications in high-throughput screening. An alternative cell viability assay that measures cellular ATP levels (termed ATP content assay) with a luminescence readout has been successfully applied in high throughput screening to identify lead compounds for a verity of targets [10], [28], [29], [30], [31]. ATP is the primary energy storage in all cells and can be used as an important marker for the functional integrity of live cells. The ATP content assay has been used to determine compound cytotoxicity in mammalian cells and more recently *Thielavia subthermophila* fungal growth [32], [33], [34]. This assay is a homogenous assay with ‘one step’ reagent addition in which no cell wash step is required. A luminescence signal is generated upon the release of cellular ATP that is detectable following a 10-minute incubation. A unique feature of the ATP content assay is that the signal-to-basal ratio is characteristically much higher in comparison to the AlamarBlue assay, partly due to low innate luminescence background generated by cells, reagents or plates. Our results have demonstrated that the new assay in the 1536-well plate format can measure the sensitivities of thousands of known drugs against *E. rostratum* in a short period of time that is not possible for conventional antibiotic susceptibility testing (AST). This new high throughput method may also be extended to other fungi or bacteria for identification of useful drugs from all known approved compounds.

Our data indicate that two potent triazole antifungal agents including posaconazole and lanoconazole (under development) may be useful as alternative agents for the treatment of infections with *E. rostratum*. The high potency and fungicidal activity of posaconazole to *E. rostratum* may suggest a role in the treatment of non-CNS infections such as septic joints that have also featured predominantly in the current outbreak and may allow prolonged oral therapy. Posaconazole has been reported to penetrate the blood brain barrier and may thus be an effective alternative for fungal infections within the central nervous system, though it does not reach reliable CNS levels compared to azoles such as voriconazole, [20].

Bithionol, an old anti-parasitic drug, is not previously known for its antifungal activity. The serum concentration of this drug has been found to be between 0.08 and 0.18 mg/l during administration and has been used successfully in the treatment of CNS *Paragonimus* infections [21], [22]. Thus, it might be a good candidate for the further clinical trials to treat CNS infections of *E. rostratum*.

Although tacrolimus, an immunosuppressive agent, showed potent activity against *E. rostratum* in this study, it would not be useful for treatment of infections with *E. rostratum* because the immunosuppression may counteract its fungicidal activity. The mechanism of action of tacrolimus has been reported through inhibition of calcineurin that, in mammalian cells, prevents transport of the nuclear factor of activated T cells (NF-ATc) to the nucleus and reduces protective IL-2 production [23]. Tacrolimus is a lipophilic agent that may have several neurotoxic effects, especially on lipid-rich white matter which is reversible after dose reduction [24], [25]. However, the potent effect of tacrolimus against *E. rostratum* suggests that anti-calcineurin compounds without these immunosuppressive properties may be of benefit against pigmented molds. This indicates that calcineurin might be a new target for drug development to treat infections with *E. rostratum*.

Floxuridine, an antimetabolite cancer drug, exhibited high nanomolar activity against *E. rostratum*. However, its cytotoxicity would likely prevent its use in treatment of infection with *E. rostratum*. Thus, the structure of this compound requires modification to reduce cytotoxicity and to enhance potency against *E. rostratum* before being applied as a therapeutic drug.

While many of the confirmed hits may not be suitable for treatment of fungal infections, their fungicidal activities may suggest new targets for future drug development after their potencies are optimized. For example, azoxystrobin, captan, pyrrolnitrin and hexachlorophene inhibit respiration [35], [36], [37], [38]; myriocin inhibits serine palmitoyltransferase [39], [40]; and thimerosal and phenylmercuric acetate are toxic organomercuries [41], [42]; while clioquinol and fenticlor have unclear mechanisms of action. Bleomycin sulfate, floxuridine and oxaliplatin inhibit DNA synthesis [43], [44], [45], which suggests that a selective fungal DNA synthesis inhibitor might be developed as a new antifungal agent against molds such as *E. rostratum*. Tribromosalan and bithionol are nuclear factor-kappa B (NF-κB) inhibitors [11]. Phenylmercuric borate is an organomercury [46], [47]. Tacrolimus binds to the immunophilin FKBP12 to inhibit calcineurin [48]. Cerivastatin sodium is an inhibitor of hydroxymethylglutaryl-coenzyme A (HMG-CoA) reductase [49]. Tyrphostin AG 879 is an inhibitor of tropomyosin-receptor-kinase (TRK) receptor [50]. Tyrphostin A9 is an inhibitor of calcium release-activated calcium (CRAC) channels [51]. Calcimycin is an ionophore [52]. The MOA & molecular targets of the other 6 compounds are yet to be elucidated. Thus, the data indicate that a rich collection of new drug targets can be identified from such a drug repurposing screen.

In conclusion, we have developed two high throughput assays using conidia and hyphae of *E. rostratum* in an ATP content assay format. A screen of 4096 compounds from an approved drug library and a pharmaceutically active compound collection using this assay led to identification and confirmation of a group of potential antifungal agents. While several triazole antifungal agents including posaconazole and lanoconazole could be used as alternative therapies for the treatment of infections with *E. rostratum*, the other confirmed compounds may implicate new drug targets for further drug development to improve their selectivity against molds such as *E. rostratum*. We believe that implementation of such a drug repurposing screen within a 7 week window provides a useful paradigm. It meets the challenges of future public health threats by enabling the rapid identification of potential therapeutic agents for infected patients and for identification of new drug development targets.

## Supporting Information

Table S1 **Activities of 26 known antifungal compounds fungicidal for both hyphae and conidia of** ***E. rostratum*** **.**
(DOCX)
