# Correction: Rapid Identification of Antifungal Compounds against *Exserohilum rostratum* Using High Throughput Drug Repurposing Screens

**DOI:** 10.1371/annotation/df5a5a46-cf30-4842-bf11-b6cee36c1f9b

**Published:** 2013-09-10

**Authors:** Wei Sun, Yoon-Dong Park, Janyce A. Sugui, Annette Fothergill, Noel Southall, Paul Shinn, John C. McKew, Kyung J. Kwon-Chung, Wei Zheng, Peter R. Williamson

The legends for Figure 6 and Figure 7 have been swapped.

Figure 6 should read:

Validation of anti-E. rostratum activities of 8 confirmed compounds in colony forming assay.

Hyphal fragment suspensions were treated with either 28 nM amphotericin B, 242 nM posaconazole, 406 nM lanoconazole, 4.38 µM itraconazole, 5.2 µM voriconazole, 12.8 µM luliconazole, 64 nM tacrolimus, 1.692 µM floxuridine or 1% DMSO (a solvent control) in PBS. Mixtures were cultured at 37°C in a shaking incubator for 24 h, then the hyphal suspensions were inoculated on Yeast Extract Peptone Dextrose (YPD) plates that were incubated for 1 day. The number of colonies on each YPD plate was counted. Results from three independent experiments were averaged; error bars show standard deviation.

doi:10.1371/journal.pone.0070506.g007

Figure 7 should read:

Distribution of molecular targets of 18 confirmed compoundss.

The antifungal activities against E. rostratum were identified for 8 other anti-infectious agents and 10 other drugs (for details, see Table 2). Their molecular targets and mechanism of action are known although their antifungal activities were not previously reported. These reported molecular targets may implicate potential new directions for anti-E. rostratum drug development.

doi:10.1371/journal.pone.0070506.g006 

