# Correction: Rapid Identification of Antifungal Compounds against *Exserohilum rostratum* Using High Throughput Drug Repurposing Screens

**DOI:** 10.1371/annotation/1d0f9e65-e7a3-41ba-960e-c4274334f436

**Published:** 2013-10-17

**Authors:** Wei Sun, Yoon-Dong Park, Janyce A. Sugui, Annette Fothergill, Noel Southall, Paul Shinn, John C. McKew, Kyung J. Kwon-Chung, Wei Zheng, Peter R. Williamson

The protein "col1agen β" appears incorrectly throughout the manuscript. The correct protein should be collagen Ⅲ.

