# Supplementary material for: Rapid Identification of Antifungal Compounds against Exserohilum rostratum Using High Throughput Drug Repurposing Screens
Source: PLoS One. 2013 Aug 21;8(8):e70506. doi: 10.1371/journal.pone.0070506 (PMC3749181; doi:10.1371/journal.pone.0070506)
Supplement: Table S1 — Activities of 26 known antifungal compounds fungicidal for both hyphae and conidia of E. rostratum . (DOCX) [file pone.0070506.s001.docx]

**Table S1.** Activities of 26 known antifungal compounds fungicidal for both hyphae and conidia of *E. rostratum*.

| Name | Hyphae | | Conidia | | SH-SY5Y | |
| --- | --- | --- | --- | --- | --- | --- |
|  | IC_50_ (µM) | % M. Res. | IC_50_ (µM) | % M. Res. | IC_50_ (µM) | % M. Res. |
| Amphotericin B | 0.014 | 84 | 0.806 | 104 | Inact. | Inact. |
| Ketoconazole | 2.38 | 74 | 3.47 | 101 | Inact. | Inact. |
| Triticonazole | 5.82 | 74 | 6.95 | 100 | Inact. | Inact. |
| Tioconazole | 5.97 | 100 | 8.55 | 101 | Inact. | Inact. |
| Terconazole | 6.12 | 100 | 9.06 | 101 | Inact. | Inact. |
| Luliconazole | 6.40 | 82 | 4.39 | 100 | Inact. | Inact. |
| Bifonazole | 11.8 | 74 | 9.35 | 64 | 46.9 | 47 |
| Econazole nitrate | 11.8 | 77 | 13.2 | 64 | Inact. | Inact. |
| Tetramethylthiuram monosulfide | 67.9 | 59 | 3.4 | 23 | Inact. | Inact. |
| Terbinafine | 34.0 | 64 | Inact. | Inact. | Inact. | Inact. |
| Azaconazole | 46.9 | 64 | Inact. | Inact. | Inact. | Inact. |
| Natamycin | 46.9 | 66 | Inact. | Inact. | Inact. | Inact. |
| Mepartricin | Inact. | Inact. | 13.2 | 80 | 37.2 | 38 |
| Griseofulvin | Inact. | Inact. | Inact. | Inact. | Inact. | Inact. |
| 2-Mercaptobenzothiazole | Inact. | Inact. | Inact. | Inact. | Inact. | Inact. |
| Flucytosine | Inact. | Inact. | Inact. | Inact. | Inact. | Inact. |
| Azaserine | Inact. | Inact. | Inact. | Inact. | Inact. | Inact. |
| Exalamide | Inact. | Inact. | Inact. | Inact. | Inact. | Inact. |
| Fluconazole | Inact. | Inact. | Inact. | Inact. | Inact. | Inact. |
| Fezatione | Inact. | Inact. | Inact. | Inact. | Inact. | Inact. |
| Fosfluconazole | Inact. | Inact. | Inact. | Inact. | Inact. | Inact. |
| Decanohydroxamic acid | Inact. | Inact. | Inact. | Inact. | Inact. | Inact. |
| Dexfosfoserine | Inact. | Inact. | Inact. | Inact. | Inact. | Inact. |
| Siccanin | Inact. | Inact. | Inact. | Inact. | Inact. | Inact. |
| Neboglamine | Inact. | Inact. | Inact. | Inact. | Inact. | Inact. |
| Tolindate | Inact. | Inact. | Inact. | Inact. | Inact. | Inact. |

Note: % Max. Resp.: % maximal response (46 µM Amphotericin B is considered as 100%); Inact. in the conidia assay or SH-SY5Y cytotoxicity assay: no significant activity at the highest compound concentration (46 µM); Inact. in the hyphae assay - no significant activity in the primary screen.
